# Crystallographic structure determination and analysis of a potential short-chain dehydrogenase/reductase (SDR) from multi-drug resistant *Acinetobacter baumannii*

**DOI:** 10.1371/journal.pone.0289992

**Published:** 2023-08-24

**Authors:** Seyed Mohammad Ghafoori, Soha Abdollahpour, Paniz Shirmast, Jade K. Forwood

**Affiliations:** 1 School of Dentistry and Medical Sciences, Charles Sturt University, Wagga, Wagga, NSW, Australia; 2 Menzies Health Institute Queensland and School of Medical Sciences, Griffith University, Gold Coast, QLD, Australia; Pohang University of Science and Technology, REPUBLIC OF KOREA

## Abstract

Bacterial antibiotic resistance remains an ever-increasing worldwide problem, requiring new approaches and enzyme targets. *Acinetobacter baumannii* is recognised as one of the most significant antibiotic-resistant bacteria, capable of carrying up to 45 different resistance genes, and new drug discovery targets for this organism is an urgent priority. Short-chain dehydrogenase/reductase enzymes are a large protein family with >60,000 members involved in numerous biosynthesis pathways. Here, we determined the structure of an SDR protein from *A*. *baumannii* and assessed the putative co-factor comparisons with previously co-crystalised enzymes and cofactors. This study provides a basis for future studies to examine these potential co-factors *in vitro*.

## Introduction

Antimicrobial resistance (AMR) is arguably one of the most critical challenges worldwide, with predictions estimating AMR infections to be a leading cause of mortality by 2050 without new treatment alternatives [1]. *Acinetobacter baumannii* is an important drug-resistant pathogen, with infection rates of ~2% of all healthcare-associated infections in the United States and Europe and ~4% in the Middle East and Asia in 2017 [2]. Despite the infection rate being lower than other gram-negative bacteria, *A*. *baumannii* exhibits high rates of multidrug resistance (MDR), that is ~45% globally and up to 70% in Latin America and the Middle East [3]. The World Health Organisation acknowledged this concern in 2017, publishing a list of the most problematic microbes with a desperate need for research and the development of new treatments, where *A*. *baumannii* was listed as a priority one, critical pathogen [4].

*Acinetobacter baumanni*, a gram-negative, non-motile, pleomorphic bacillus, is an opportunist and robust species with a remarkable ability to survive, adapt, and accumulate AMR. The capacity for AMR in *A*. *baumanni* stems from both natural and acquired features. *A*. *baumannii* has a minimal number of small porins in the outer membrane, resulting in a low permeability to antibiotics [5]. The bacterium also expresses efflux pumps, assisting resistance to a broad range of antibiotics [5], and harbours a substantial resistance island, enabling expression of up to 45 resistance genes [6,7]. Moreover, the bacterium can acquire resistance genes through genetic elements such as transposons and plasmids [8]. These genes provide *A*. *baumanni* the ability to utilise different AMR enzymes such as AmpC cephalosporinases, OXA carbapenemases, KPC serine carbapenemases, metallo-carbapenemases, and aminoglycosides, and form the basis for an extremely drug resistant pathogen [9].

Within a clinical setting, *A*. *baumannii* has the capacity for both nosocomial and community-acquired infections leading to meningitis, septicaemia, pneumonia, wound and urinary infections, with increased risk through the use of catheters and ventilators in immunocompromised and elderly patients [10]. Endotracheal tubes and mechanical ventilators present two common modes for transmission of *Acinetobacter* in hospitals due to the ability of this bacteria to adhere to plastic and form biofilms [11]. Additionally, use of central venous or urinary catheters present the most common cause of bloodstream infections and urinary tract infections by *A*. *baumannii* [11]. Postsurgical infections such as wound infections, endocarditis, meningitis, and osteomyelitis represent other types of infection caused by *A*. *baumannii* [12–14]. Community-acquired infections are also reported and occur mainly in warm, humid, and tropical environments, especially in the east of Asia, Australia, and Oceania [15,16]. Underlying medical conditions such as kidney disease, cancer, and diabetes present as predispositions to *A*. *baumannii* community-acquired infections including acute pneumonia [17].

*A*. *baumannii* has displayed resistance and reduced sensitivity to antimicrobials including last resort carbapenem, polymyxins, and tigecyclines [18]. Due to the increasing resistance rate against antibiotics, the generation of new therapies are under investigation to overcome the permeability, efflux, and enzymatic resistance mechanisms in this highly adaptable pathogen. This includes use of antimicrobial peptides, phototherapy, bacteriophages, active and passive vaccination, and trace metal sequestration towards generating new therapies against AMR strains [9,19,20]. The short-chain dehydrogenase/reductase family, consisting of 60,000 documented enzymes, [21] carry out the catalysis of oxidation/reduction reactions using either nicotinamide adenine dinucleotide (NADH) or nicotinamide adenine dinucleotide phosphate (NADPH) as a co-factor [22]. SDR families share several standard features, despite sharing low sequence similarities (less than 30%), including the Rossman fold domain and the active site, consisting of a highly conserved tyrosine, lysine, serine, and often an asparagine [23]. Here, an SDR family member from *A*. *baumannii* (Uniprot ID: A0A0M3FP68) was cloned, expressed, purified, and crystallised. Crystals diffracted to 2.4 Å, permitting structure determination. Based on the structure and bioinformatics analysis, potential cofactors were investigated.

## Materials and methods

### Recombinant protein expression, purification, and structure determination

The putative SDR sequence (Uniprot ID: A0A0M3FP68) from *A*. *baumannii* was codon-optimized, cloned into a pMCSG21 expression vector, expressed using auto-induction method, and purified as previously described [24]. In summary, competent pLysS *E*. *coli* BL21(DE3) cells were transfomed with plasmid using the heat shock method. Protein expression was carried out for 36 h at room temperature, and cells were harvested by centrifugation. Following two freeze-thaw cycles, cells were treated with DNase (∼0.025mg/mL) and lysozyme (∼2mg/mL). After centrifugation, the soluble extract was injected onto a pre-equilibrated GE HisTrap 5mL column, washed with 10 column volumes of His A buffer (50mM phosphate buffer, 300mM sodium chloride, 20mM imidazole, pH 8.0) and eluted using His B buffer (50mM phosphate buffer, 300mM sodium chloride, 500mM imidazole, pH 8.0). Fractions containing protein were pooled and treated with tobacco etch virus protease at 4°C for ~14h. Size exclusion chromatography was performed using Superdex 200pg 26/600 column using Tris-Buffered Saline (50mM tris, 125mM sodium chloride, pH 8.0). Purified protein was concentrated and stored at -80°C for future assays [25]. Crystallization trials were performed using the hanging-drop vapour diffusion method using commercially available screening kits in 48 well plates. Each drop consisted of 1.5 μL of reservoir and 1.5 μL of the protein with the final concentration of 1.7 mg/mL suspended over 300 μL of the reservoir. Rod-shaped crystals were harvested from a reservoir solution containing 0.2 M lithium chloride 0.1 M Tris pH 8.0, 20% (v/v) polyethylene glycol 8,000, flash-cooled in liquid nitrogen and sent to MX2 beamline at the Australian Synchrotron for X-ray diffraction measurements [26]. The x-ray diffraction data was collected on an EigerX 16M detector, indexed in iMosflm [27], merged and scaled in Aimless [28], phased by molecular replacement using Phaser [29], refined using Phenix [30], and modelled in Coot [31]. To compare our structure with previously determined SDRs, structural alignments were performed using the DALI server [32], and the structures superimposed using PyMol software (Version 2.3.3) [33]. The PDBsum server was used to examine binding interfaces and co-factor binding site residues [34].

## Results and discussion

**Fig 1:**
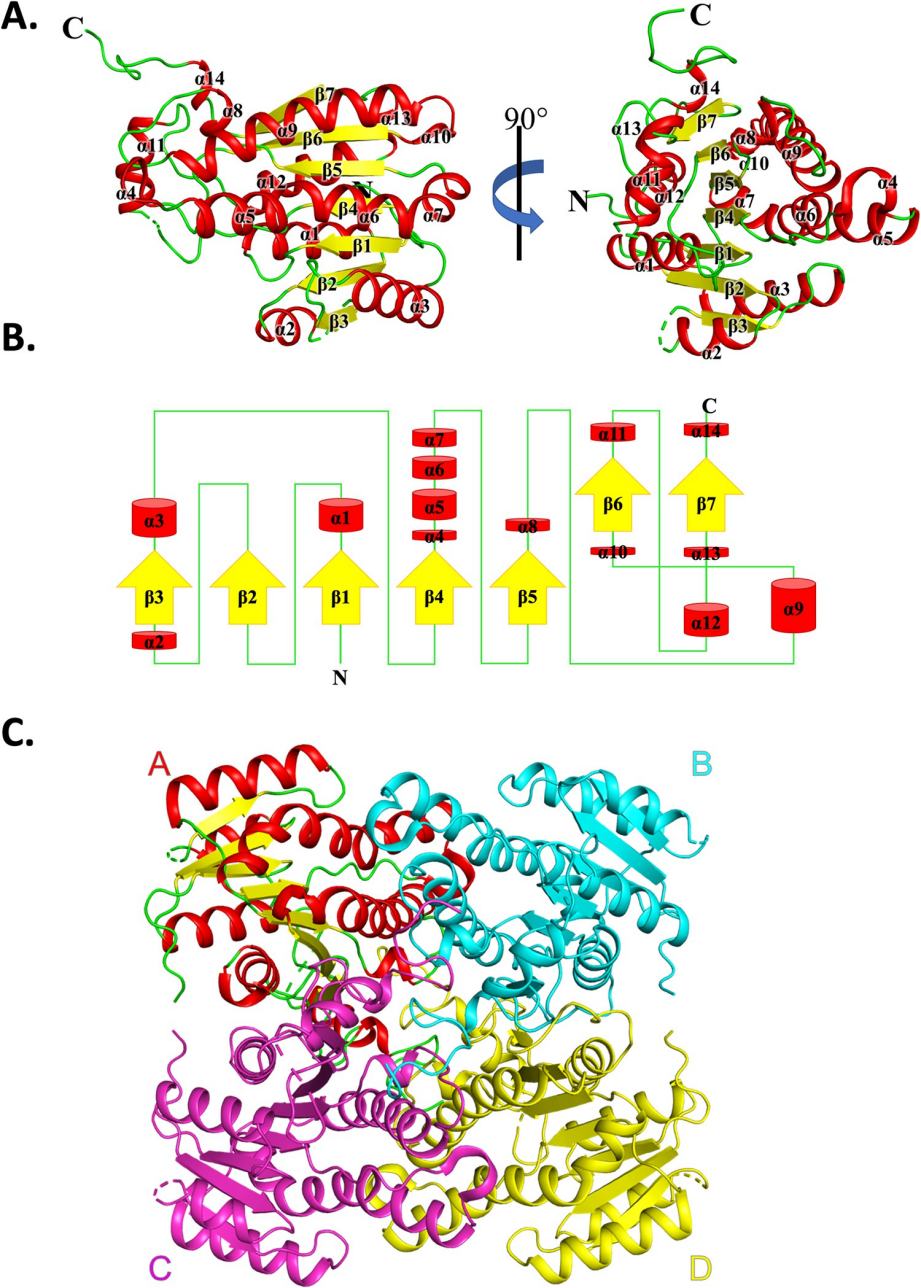
(A) Tertiary structure of the SDR protomer. The central β-sheet (yellow) is sandwiched by two groups of α-helices (red). (B) Topological map of the monomer with colour coding maintained as in panel A. (C) Homo-tetrameric structure of the *A*. *baumannii* SDR protein. In protomer A, secondary structures have been shown using yellow for β-sheets, red for α-helices, and green for connecting loops. Three types of interfaces in the tetramer are identified as A/B, A/C, and A/D.

*A*. *baumannii* has been identified as a critical pathogen in desperate need of new treatment options due to the alarming rate of increasing resistance to all classes of antibiotics, including last resort carbapenem [9]. Here we selected an *A*. *baumannii* SDR enzyme with no known structure, and for which the closest sequence identity to any known protein structure in the Protein Data Bank is ~37% to expand the repertoire of previously determined SDR proteins from this important bacterium [25,35,36]. The protein structure was determined by X-ray crystallography and compared with related SDRs to provide a platform for further studies. During protein purification, the purity at each step was assessed by SDS-PAGE, and the sharp elution peak from the size exclusion column corresponded to a molecular weight of about 100 kDa, suggesting the enzyme was a tetramer (27 kDa protomer). Crystal X-ray diffraction was collected at the Australian Synchrotron MX2 macromolecular crystallography beamline [26,37]. The diffraction dataset was indexed and integrated in C 2 2 2_1_ with iMosflm and scaled and merged in Aimless to 2.4 Å (Table 1). The Matthews coefficient was 2.29 (corresponding to a crystal solvent content of 46.2%, suggested that the asymmetric unit was most likely comprised of two protomers. Data was phased using PhaserMR [29] using molecular replacement with a monomer of oxidoreductase TT0321 from *Thermus thermophilus* (PDB: 2D1Y; 37% sequence identity to our SDR enzyme). Four molecules were placed in the asymmetric unit, and rebuilding/refinement was performed to an R/R_free_ of 19.0 and 22.6% respectively. Due to poor density, residues 49–53 and 189–196 of chains A and C, and residues 50–51 and 189–197 of chains B and D were unable to be modelled, and density for the N-terminal methionine was not of sufficient quality to model this residue. After structural elucidation it was discovered that Gln69 was missing, and investigation revealed this error was introduced in the original design of the plasmid. Fortunately, overlay of the experimentally determined structure with the Gln69 deletion and the alpha fold prediction without the Gln69 deletion showed that the overall structure was not affected, and it overlayed perfectly with previously determined SDR structures. Each monomer contains a central β -sheet (β1- β7), sandwiched between two groups of α- helices (α1-α2-α12-α13 and α3-α5-α6-α7-α8-α9-α10), with an overall topology of β1-α1-β2-α2-β3-α3-β4-α4-α5-α6-α7-β5-α8-α9-α10-β6-α11-α12-α13-β7-α14 (Fig 1A and 1B).

**Table 1:** Data and refinement statistics.

Specifications	PDB ID: 8GBB
Wavelength (Å)	0.9537
Resolution range (Å)	29.06–2.4 (2.486–2.4)
Space group	*C*222_1_
Cell dimensions	
a, b, c (Å)	67.43 109.46 136.92
a = b = g (°)	90
Total reflections	576,618 (51732)
Unique reflections	20,190 (1976)
Multiplicity	28.6 (26.0)
Completeness (%)	99.72 (99.35)
Mean I/σ (I)	31.50 (4.67)
Wilson B-factor (Å^2^)	24.95
R_meas_	0.8757 (2.149)
R_pim_	0.164 (0.4205)
CC1/2	0.89 (0.694)
Reflections used in refinement	20171 (1974)
Reflections used for R-free	20149 (1974)
R_work_/R_free_	0.1922 / 0.2303
Number of atoms	4043
macromolecules	3845
solvent	198
Protein residues	520
RMSD bonds (Å)	0.004
RMSD angles (°)	0.58
Ramachandran favoured (%)	96.71
Ramachandran allowed (%)	3.29
Ramachandran outliers (%)	0.00
Clashscore	1.70
Average B-factor (Å^2^)	31.44
B-factor macromolecules (Å^2^)	31.52
B-factor solvent (Å^2^)	29.95

*Statistics for highest resolution shell are shown in parentheses.

To examine key interactions that mediate binding at the tetramer interface, the crystal structure was analysed using Proteins, Interfaces, Structures and Assemblies (PISA) [38,39]. Three interfaces were identified, and labelled A/B, A/C, and A/D (Fig 1C; S1 Table). The A/B interface was mediated by 29 hydrogen bonds and 8 salt bridges and comprised a buried surface area of 1635.8(A)/1642.9(B) Å^2^. The A/C interface was mediated by 16 hydrogen bonds and buried a surface area of 1408.6(A)/1407.7(B) Å^2^. The A/D interface was mediated by 11 hydrogen bonds and 2 salt bridges and buried an interface of 914.4(A)/906.5(B) Å^2^.

**Fig 2:**
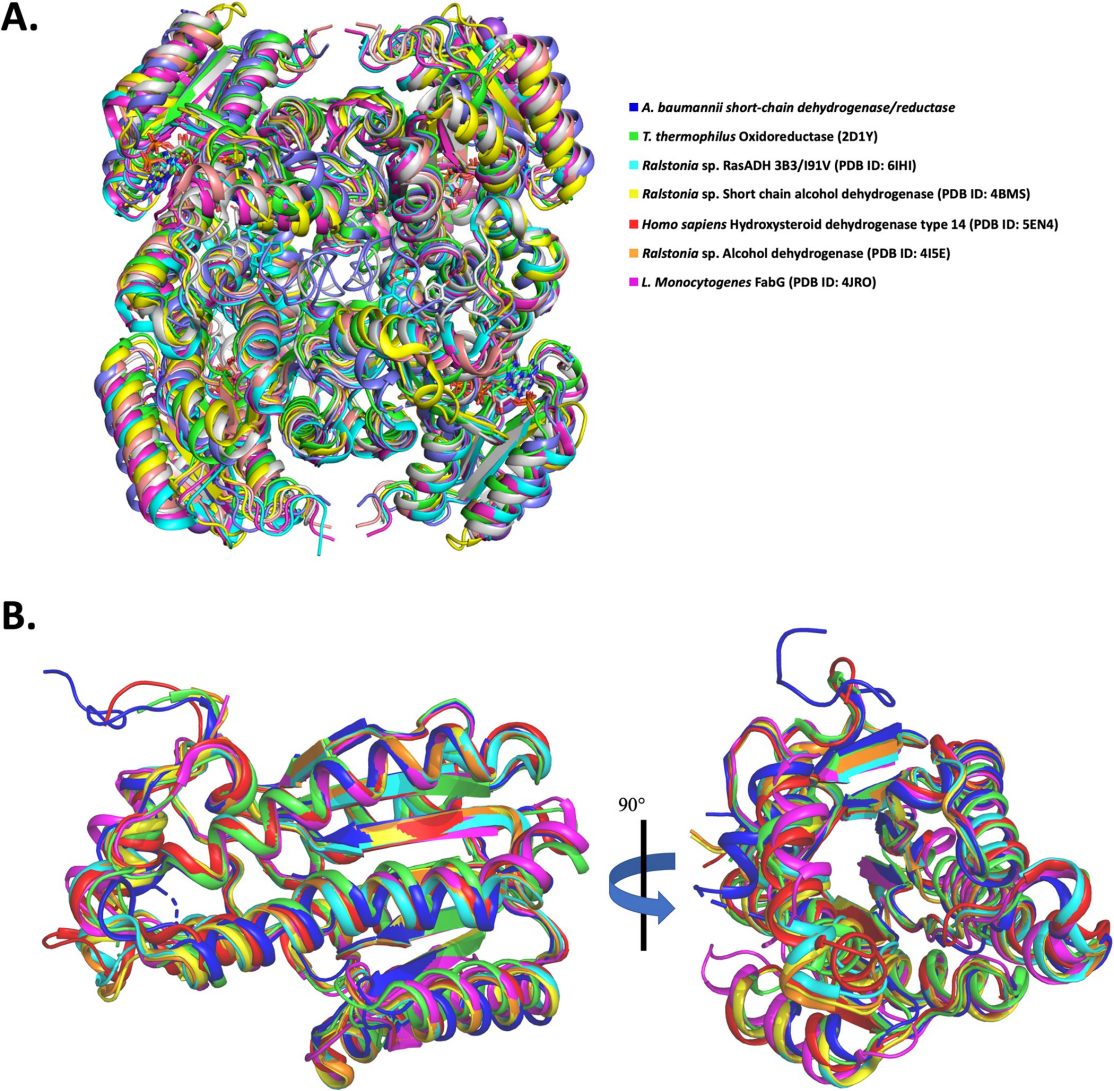
Superimposed structure of the *A*. *baumannii* SDR and six structurally similar SDR enzymes. (A) Superimposition of tetrameric structures of the enzymes, (B) Superimposition of protomers at zero degrees and 90 degrees. Each enzyme shown from the PDB is colour coded according to the legend.

**Fig 3:**
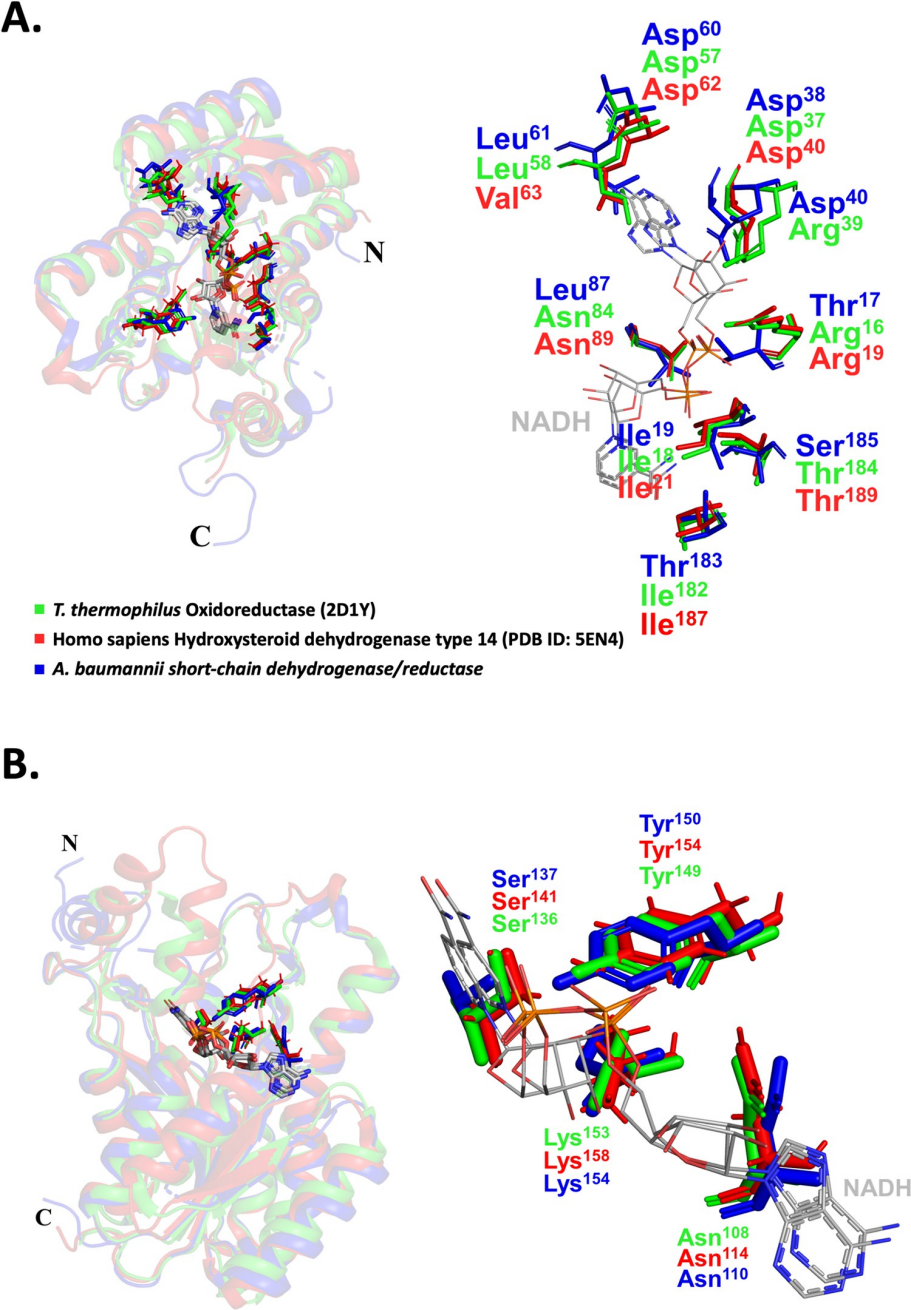
Comparison of our putative SDR enzyme from *A*. *baumannii* (dark blue) with SDR enzymes containing the NADH co-factor from *Thermus thermophilus* (green) and *Homo sapiens* (red). Co-factors are coloured in grey. (A) Position of the co-factor binding site residues (left) and zoomed view (right), and (B) catalytic residues in the cavity (left) and zoomed view (right). Colour coding is the same in both panels.

A structural alignment was performed using the DALI server to identify the most similar structures deposited to the Protein Data Bank (Table 2) [40]. We superimposed our structure (dark blue) on six of the most structurally similar SDR enzymes, confirming a high structural similarity (Fig 2). Since SDR enzymes utilize either NADH or NADPH as a cofactor, these enzymes can copurify with NADH or NADPH (sourced from the bacteria), be cocrystallised with exogenously added cofactors, or in other cases, don’t bind cofactors *in vitro* due to crystallographic packing or other forms of regulation [25,36,41,42]. The structure presented in this study presented no evidence of the cofactor (or substrate) bound in the well described binding pocket. Moreover, addition of exogenous cofactors added to the protein prior to crystallisation did not reveal density to support the modelling. Therefore to examine whether the enzyme investigated in this study may potentially recognise either of these cofactors, a structural alignment was performed with SDR enzymes bound to either NADH or NADPH. Firstly, we superimposed SDR enzymes containing NAD, including an oxidoreductase from the *T*. *thermophilis* species (green) and a hydroxysteroid dehydrogenase from *Homo sapiens* (red) (Fig 3). The residues that hydrogen bond with NAD in the *T*. *thermophilis* structure were Arg16, Ile18, Asp37, Arg39, Asp57, Leu58, Asn84, Ile182, and Thr184, four of which exist at the same position as the SDR in this study (Ile19, Asp38, Asp60, Leu61). In the *Homo sapiens* SDR structure Arg19, Ile21, Asp40, Asp62, Val63, Asn89, Ile187, Thr189 hydrogen bonded with NAD, of which three were conserved in the SDR of this study (Asp60, Asp38, Ile19). All four catalytic residues, previously characterised to have catalytic function in SDRs, are conserved in the SDR structure determined in this study (Asn108, Ser136, Tyr150, Lys153) (Fig 3B).

**Table 2:** List of structurally similar enzymes for superimposition. To identify similar enzymes, a structural search was performed using the DALI server (http://ekhidna2.biocenter.helsinki.fi/dali/). Of six similar enzymes (with RMSD below), two were chosen for structural analysis.

No.	PDB ID	Enzyme name	Organism	RMSD (Å)	No. residues	identity (%)	Ligand
1	2D1Y	Oxidoreductase	*Thermus thermophilus*	1.4	240	38	NADH
2	6IHI	RasADH 3B3/I91V	*Ralstonia* sp.	1.5	235	28	NADPH
3	4BMS	Short-chain alcohol dehydrogenase	*Ralstonia* sp.	1.7	247	27	NADPH
4	4JRO	FabG	*Listeria monocytogenes*	1.9	244	31	NADPH
5	4I5E	Alcohol dehydrogenase	*Ralstonia* sp.	1.7	249	27	NADPH
6	5EN4	Hydroxysteroid dehydrogenase	Homo sapiens	1.8	251	32	NADH

**Fig 4:**
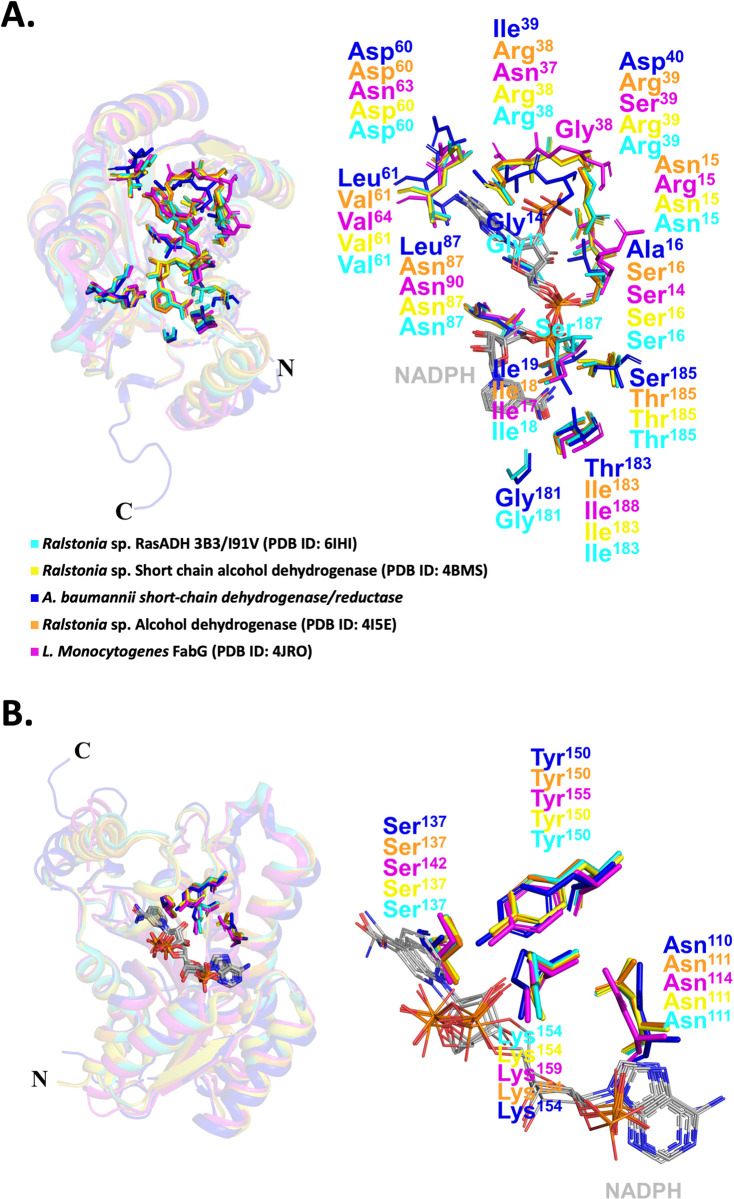
Comparison of our putative SDR co-factor (NADPH) acceptor site from *A*. *baumannii* (dark blue) with RasADH 3B3/I91V from *Ralstonia* sp. (cyan), short chain alcohol dehydrogenase from *Ralstonia* sp.(yellow), alcohol dehydrogenase from *Ralstonia* sp. (orange), and FabG from *Listeria monocytogenes* (magenta). Co-factors are coloured in grey. (A) Position of the acceptor site residues in enzyme (left) and zoomed view (right), and (B) catalytic residues in the enzyme cleft (left) and zoomed view (right). Colour coding are same in both panels.

The second comparison was performed between the SDR of this study (dark blue) and SDR enzymes containing the NADPH cofactor; PDB 4BMS in yellow; PDB 4I5E in orange; PDB 6IHI in cyan and PDB 4JRO in purple (Fig 4, and Table 2 for details). While all typical catalytic residues are conserved (Asn110, Ser137, Tyr150, and Lys154), only two residues (Asp60 and Ile19) are conserved at the co-factor binding site in the SDR within this study. This may suggest that the co-factor for SDR enzyme in this study is more likely to be NADH, however this needs to be determined experimentally once the substrate has been determined.

**Fig 5:**
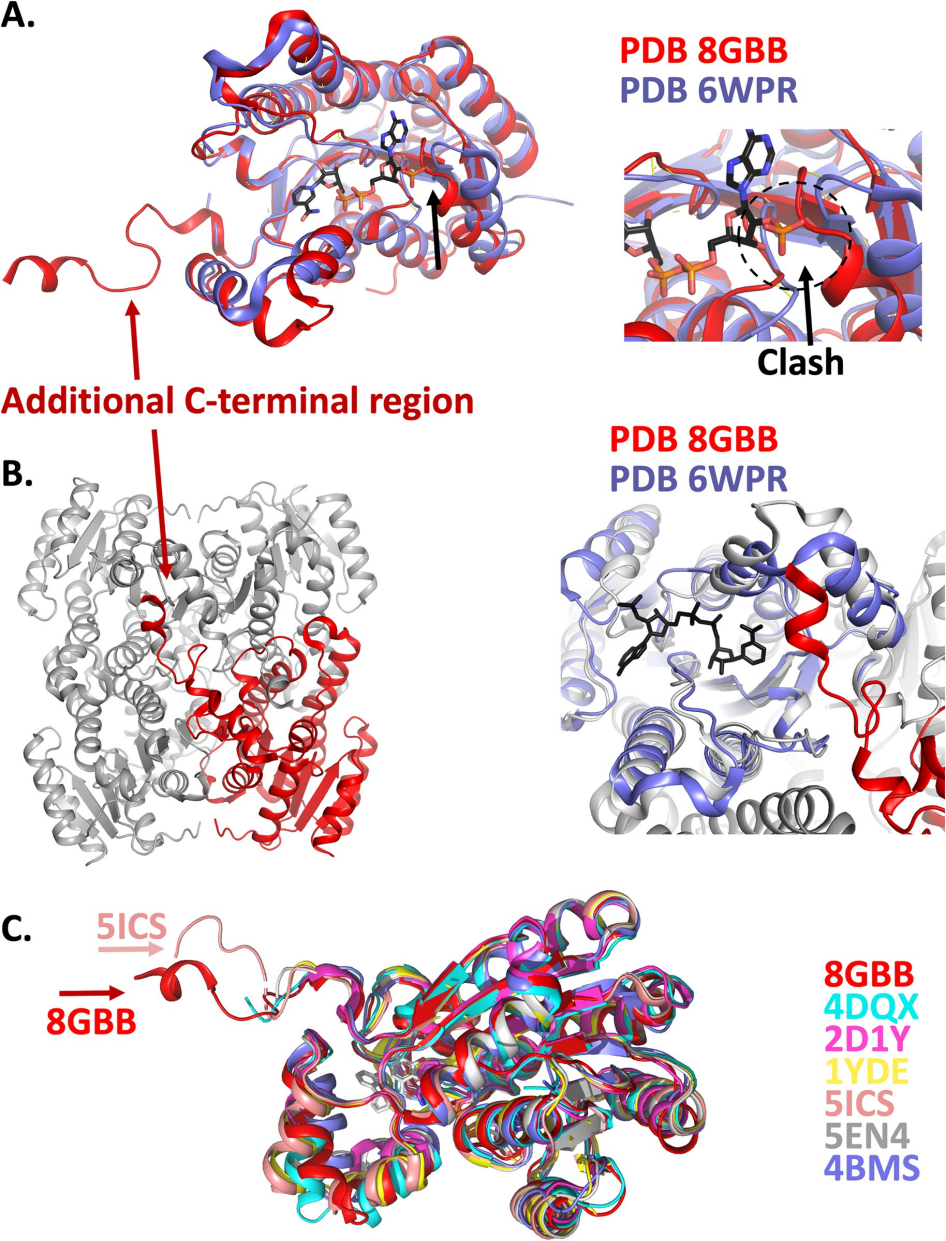
A. Structural alignment of the SDR investigated in this study (PDB 8GBB; coloured red) with an SDR crystallized with NADPH (in black sticks) (PDB 6WPR; coloured blue). The phosphate moiety of NADPH clashes with a loop on ^38^DIDAK^42^ (see right inset). B. The C-terminal region of the SDR in this study, spanning residues ^246^VMGPEGLQPAIPRLAE^261^ extends into the diagonally adjacent protomer. Binding is stablised by interaction with loop regions within 186–248 that play a role in cofactor and substrate binding. The inset shows the positioning of this C-terminal extension in relation to the cofactor from PDB 6WPR. C. Superposition of structurally conserved SDR’s reveal that the C-terminal extension observed in this structure is unusual, but not completely unique. The structure of the SDR enzyme from PDB 5ICS reveals a similar C-terminal extension that also extends into an adjacent protomer.

In further support that NADH is more likely to be the cofactor rather than NADPH, superposition revealed a clash between the phosphate of NADPH and the loop between ^38^DIDAK^42^ (Fig 5A). This analysis also revealed that uniquely, the C-terminus of the SDR in this study, spanning residues ^246^VMGPEGLQPAIPRLAE^261^ extended diagonally and bound the adjacent protomer by interacting with loop regions 186–248 (Fig 5B), a region that plays a role in cofactor and substrate binding. Specifically, this interaction was mediated by Val^246^ hydrogen bonding with Ala^143^, Glu^250^ hydrogen bonding with Gln^144^, and Ala^260^ hydrogen bonding with Lys^198^, as well as a salt bridge interaction between Arg^258^ and Asp^205^. To examine if this C-terminal extension was conserved in other SDR’s we performed a DALI structural analysis, and compared these regions. Extensive examination of the most structurally conserved structures revealed that only one other SDR structure contained a similar extension (Fig 5C) (PDB 5ICS; [43]). Whilst tempting to speculate on the potential role of this region to regulate substrate and/or cofactor binding, extensive experimental evidence is required to assess this, and would firstly require knowledge of the substrate.

**Fig 6:**
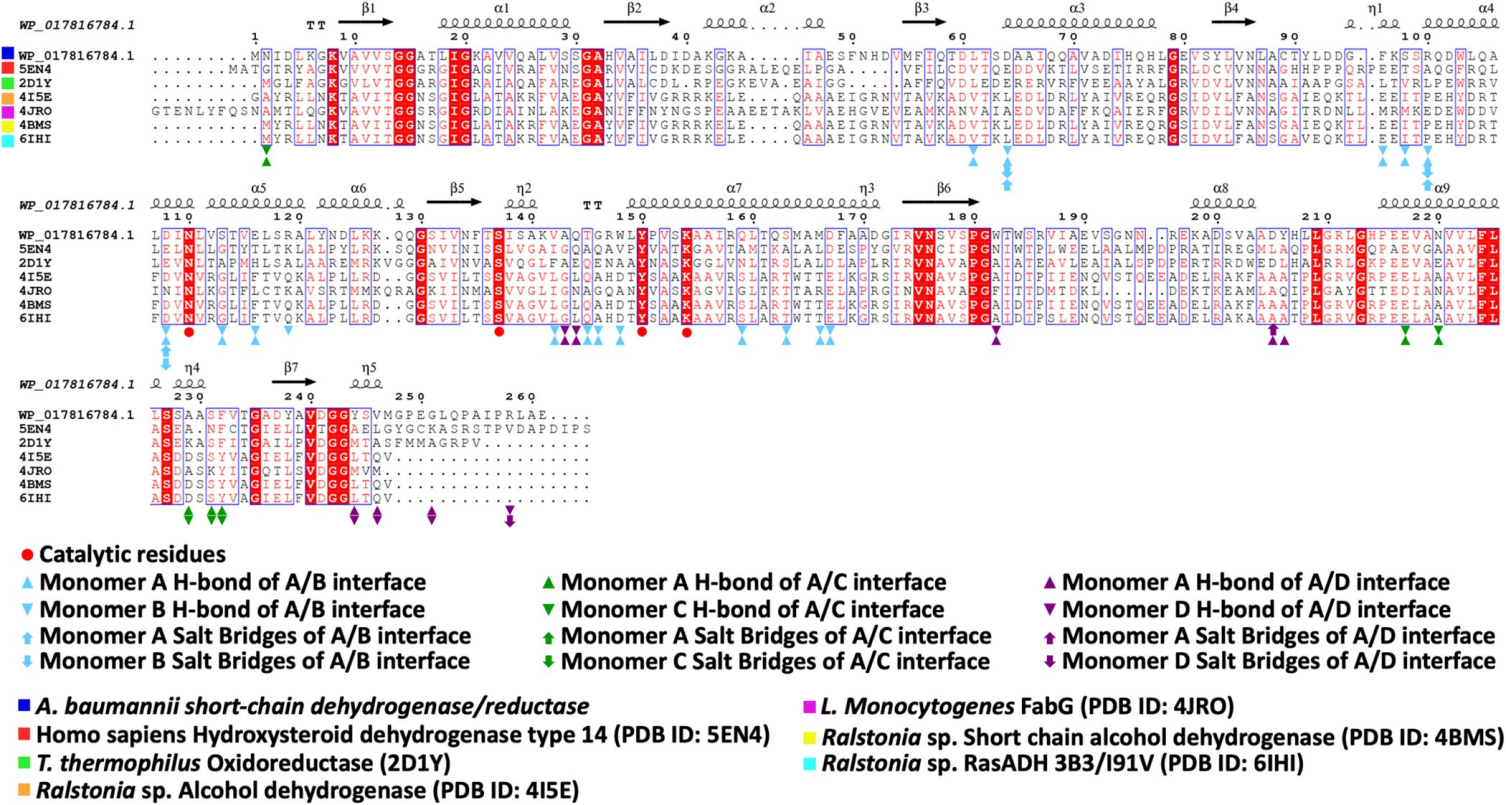
Multiple sequence alignment of *A*. *baumannii* putative SDR with various similar SDRs from other organisms. The secondary structural elements of the *A*. *baumannii* enzyme (PDB ID: 8GBB) are shown above the multiple sequence alignment. Conserved residues are shown in red. Four catalytic residues are denoted with red circles. Light blue, dark green and purple symbols indicate interfacial residues that form H-bonds or salt bridges between protomers in three different interfaces where residues responsible for hydrogen bonds from one monomer are indicated with triangles and residues of the second monomer are shown as inverted triangles. Also, residues responsible for salt bridges from one monomer are indicated with up arrow and residues of the second monomer are shown as inverted arrow. Protein sequences within the alignment include: *A*. *baumannii* SDR enzyme (NCBI Accession WP_017816784), Homo sapiens Hydroxysteroid dehydrogenase type 14 (PDB ID: 5EN4), *T*. *thermophilus* Oxidoreductase (2D1Y), *Ralstonia* sp. Alcohol dehydrogenase (PDB ID: 4I5E), *L*. *Monocytogenes* FabG (PDB ID: 4JRO), *Ralstonia* sp. Short chain alcohol dehydrogenase (PDB ID: 4BMS), and *Ralstonia* sp. RasADH 3B3/I91V (PDB ID: 6IHI). The multiple sequence alignment was generated with ESpript (https://espript.ibcp.fr/ESPript/ESPript/).

To investigate the potential importance of residues within the SDR, we performed a graphical alignment using the Clustal Omega online and ESpript server [44] between all amino acid sequences (Fig 6). While the similarity is not high between all sequences, four catalytic residues (Asn, Ser, Tyr, and Lys) are highly conserved in all sequences (Fig 6). Moreover, several regions have been reported as conserved motifs within classical SDRs enzymes [45], and we found seven reported motifs of the classical SDRs in our enzyme (Table 3 and Fig 6). Whilst these results suggest the enzyme is likely to be an SDR, experimental evidence is required.

**Table 3:** Comparison of reported conserved motifs in classical SDRs enzymes and equivalent motifs in our enzyme. Abbreviations are as follow: “c” charged residue, “h” hydrophobic residue, “p” polar residue, “x” any residue. Capital characters show single-letter amino acid codes. Starting and ending number of each motif is written at the start/end of the motif.

#	Reported motifs	Equivalent motifs on our SDR
1	TG xxx GhG	13-SGG AT LIG-21
2	Dhx[cp]	60-D LTS-63
3	G xh D hhh NNAGh	79-G EV S YLV N L ACT-90
4	hNhxG	109-I N LV S-113
5	GxhhxhSSh	130-G SIV NFT S I-138
6	Yx[AS][ST]K	150-Y P V SK-154
7	h[KR]h[NS]xhxPGxxxT	173-IR V N SVS PG WTW S-185

## Conclusion

In the present study, we describe the crystal structure of a putative SDR protein from *A*. *baumannii*. The structure provides an ideal platform for *in vitro* and *in silico* studies. Overall, the high-resolution structure of this enzyme, and analysis thereof, provides an ideal basis for understanding the structure-function relationship of SDRs and design of small molecule inhibitors.

## Supporting information

S1 Table A/B, A/C and A/D interfaces interactions in our SDR enzyme. Different number of hydrogen bonds, salt bridges and interface area can be seen in different interfaces.
(PDF)
