# Supplementary material for: Crystallographic structure determination and analysis of a potential short-chain dehydrogenase/reductase (SDR) from multi-drug resistant Acinetobacter baumannii
Source: PLoS One. 2023 Aug 24;18(8):e0289992. doi: 10.1371/journal.pone.0289992 (PMC10449147; doi:10.1371/journal.pone.0289992)
Supplement: S1 Table — Different number of hydrogen bonds, salt bridges and interface area can be seen in different interfaces. (PDF) [file pone.0289992.s001.pdf]

S1 Table: A/B, A/C and A/D interfaces interactions in our SDR enzyme. Different number of hydrogen bonds, salt bridges and interface area can be seen in different interfaces

	A/B Interfaces			A/C Interfaces			A/D Interfaces		
	Number of H-bonds: 29			Number of H-bonds: 16			Number of H-bonds: 11		
	Number of Salt bonds: 8			Number of Salt bonds: 0			Number of Salt bonds: 2		
	Interface area [Å²]: 1635.8/1642.9			Interface area [Å²]: 1408.6/1407.7			Interface area [Å²]: 914.4/906.5		
	Hydrogen Bonds								
#	Chain A	Dist. [Å]	Chain B	Chain A	Dist. [Å]	Chain C	Chain A	Dist. [Å]	Chain D
1	A:ARG 100[NH2]	2.95	B:LEU 61[O]	A:ASN 2[N]	3.58	C:ASN 2[O]	A:ALA 143[N]	3.05	D:VAL 246[O]
2	A:ARG 100[NE]	3.22	B:ASP 64[OD1]	A:ASN 2[ND2]	3.21	C:ASN 2[O]	A:GLN 144[NE2]	3.05	D:GLU 250[OE2]
3	A:ARG 100[NH2]	2.90	B:ASP 64[OD1]	A:PHE 232[N]	3.69	C:GLU 217[OE2]	A:TRP 182[NE1]	3.00	D:GLU 250[OE2]
4	A:ARG 119[NH2]	2.21	B:PHE 96[O]	A:SER 231[OG]	2.54	C:GLU 217[OE2]	A:TYR 244[OH]	2.77	D:GLU 250[OE2]
5	A:ARG 119[NH2]	3.11	B:SER 98[O]	A:ASN 220[ND2]	3.31	C:ALA 229[O]	A:ASP 205[OD2]	3.19	D:ARG 258[NH2]
6	A:ARG 100[NH1]	2.73	B:ASP 108[OD1]	A:GLY 242[N]	2.91	C:PHE 232[O]	A:ASP 205[OD2]	2.99	D:ARG 258[NH1]
7	A:ARG 100[NH1]	3.42	B:SER 113[OG]	A:ASP 241[N]	3.61	C:PHE 232[O]	A:TYR 206[OH]	2.95	D:ARG 258[NH1]
8	A:ARG 100[N]	3.72	B:GLU 116[OE1]	A:TYR 238[OH]	3.02	C:ALA 239[O]	A:VAL 246[O]	2.84	D:ALA 143[N]
9	A:GLN 159[NE2]	3.54	B:VAL 142[O]	A:ASN 2[O]	3.58	C:ASN 2[N]	A:GLU 250[OE2]	2.84	D:GLN 144[NE2]
10	A:TRP 148[NE1]	3.70	B:SER 163[O]	A:ASN 2[O]	3.21	C:ASN 2[ND2]	A:GLU 250[OE2]	3.08	D:TRP 182[NE1]
11	A:THR 145[N]	3.24	B:MET 166[SD]	A:GLU 217[OE2]	3.69	C:PHE 232[N]	A:GLU 250[OE2]	2.92	D:TYR 244[OH]
12	A:THR 145[OG1]	3.08	B:ASP 167[OD1]	A:GLU 217[OE2]	2.54	C:SER 231[OG]			
13	A:GLY 146[N]	3.11	B:ASP 167[OD2]	A:ALA 229[O]	3.31	C:ASN 220[ND2]			
14	A:TRP 148[NE1]	2.88	B:ASP 167[OD2]	A:PHE 232[O]	3.61	C:ASP 241[N]			
15	A:LEU 61[O]	2.85	B:ARG 100[NH2]	A:PHE 232[O]	2.91	C:GLY 242[N]			
16	A:ASP 64[OD1]	2.94	B:ARG 100[NE]	A:ALA 239[O]	3.02	C:TYR 238[OH]			
17	A:ASP 64[OD1]	2.88	B:ARG 100[NH2]						
18	A:PHE 96[O]	2.31	B:ARG 119[NH2]						
19	A:SER 98[O]	3.03	B:ARG 119[NH2]						
20	A:ASP 108[OD1]	2.65	B:ARG 100[NH1]						
21	A:SER 113[OG]	3.28	B:ARG 100[NH1]						
22	A:GLU 116[OE1]	3.83	B:ARG 100[N]						
23	A:VAL 142[O]	3.35	B:GLN 159[NE2]						
24	A:SER 163[O]	3.69	B:TRP 148[NE1]						
25	A:MET 166[SD]	3.13	B:THR 145[N]						
26	A:ASP 167[OD1]	3.06	B:THR 145[OG1]						
27	A:ASP 167[OD1]	3.48	B:GLY 146[N]						
28	A:ASP 167[OD2]	2.85	B:TRP 148[NE1]						
29	A:ASP 167[OD2]	3.16	B:GLY 146[N]						

	Salt Bonds								
	A/B Interfaces			A/C Interfaces			A/D Interfaces		
#	Chain A	Dist. [Å]	Chain B	Chain A	Dist. [Å]	Chain C	Chain A	Dist. [Å]	Chain D
1	A:ARG 100[NE]	3.22	B:ASP 64[OD1]				A:ASP 205[OD2]	3.19	D:ARG 258[NH2]
2	A:ARG 100[NH2]	2.90	B:ASP 64[OD1]				A:ASP 205[OD2]	2.99	D:ARG 258[NH1]
3	A:ARG 100[NH1]	2.73	B:ASP 108[OD1]						
4	A:ARG 100[NH1]	3.52	B:ASP 108[OD2]						
5	A:ASP 64[OD1]	2.94	B:ARG 100[NE]						
6	A:ASP 64[OD1]	2.88	B:ARG 100[NH2]						
7	A:ASP 108[OD1]	2.65	B:ARG 100[NH1]						
8	A:ASP 108[OD2]	3.40	B:ARG 100[NH1]						
